# Quantitative trait locus mapping identifies the *Gpnmb* gene as a modifier of mouse macrophage lysosome function

**DOI:** 10.1038/s41598-021-89800-5

**Published:** 2021-05-13

**Authors:** Peggy Robinet, Brian Ritchey, Shuhui Wang Lorkowski, Alexander M. Alzayed, Sophia DeGeorgia, Eve Schodowski, C. Alicia Traughber, Jonathan D. Smith

**Affiliations:** 1 Department of Cardiovascular and Metabolic Sciences, Lerner Research Institute, Cleveland Clinic, Cleveland, OH 44195 USA; 2 Department of Molecular Medicine, Cleveland Clinic Lerner College of Medicine of Case Western Reserve University, Cleveland, OH 44195 USA; 3 Present Address: ProEd Communications, Inc., Beachwood, OH 44122 USA

**Keywords:** Biochemistry, Cell biology, Genetics

## Abstract

We have previously shown that the DBA/2J versus AKR/J mouse strain is associated with decreased autophagy-mediated lysosomal hydrolysis of cholesterol esters. Our objective was to determine differences in lysosome function in AKR/J and DBA/2J macrophages, and identify the responsible genes. Using a novel dual-labeled indicator of lysosome function, DBA/2J versus AKR/J bone marrow derived macrophages had significantly decreased lysosome function. We performed quantitative trait loci mapping of lysosome function in bone marrow macrophages from an AKR/J × DBA/2J strain intercross. Four distinct lysosome function loci were identified, which we named macrophage lysosome function modifier (*Mlfm*) *Mlfm1* through *Mlfm4*. The strongest locus *Mlfm1* harbors the *Gpnmb* gene, which has been shown to recruit autophagy protein light chain 3 to autophagosomes for lysosome fusion. The parental DBA/2J strain has a nonsense variant in *Gpnmb*. siRNA knockdown of *Gpnmb* in AKR/J macrophages decreased lysosome function, and *Gpnmb* deletion through CRISP/Cas9 editing in RAW 264.7 mouse macrophages also demonstrated a similar result. Furthermore, a DBA/2 substrain, called DBA/2J-Gpnmb+/SjJ, contains the wildtype *Gpnmb* gene, and macrophages from this *Gpnmb*-preserved DBA/2 substrain exhibited recovered lysosome function. In conclusion, we identified *Gpnmb* as a causal modifier gene of lysosome function in this strain pair.

## Introduction

Atherosclerosis is a chronic inflammatory disease characterized by the subendothelial accumulation of cholesterol-laden macrophages. Macrophages store excess cholesterol as cholesterol esters (CEs) in lipid droplets, and CE must be hydrolyzed to free cholesterol (FC) to facilitate its efflux, the first step in the reverse cholesterol transport pathway that can promote plaque regression^1^. Although CE hydrolysis was originally thought to be mediated by cytoplasmic neutral CE hydrolases, it has been demonstrated lysosomal acid lipase plays a significant role in macrophage CE hydrolysis^2^. In this new pathway, lipid droplets are delivered to lysosomes via autophagy, where lysosomal acid lipase hydrolyzes CE to generate FC for cholesterol efflux^2^.

Aortic root atherosclerosis lesion area in apoE-deficient mice is modified by genetic background, with DBA/2J mice having 10-fold larger aortic lesions than AKR/J mice^3^. In cholesterol-loaded bone marrow-derived macrophages (BMDMs) from these two strains, DBA/2J cells accumulate more CE while AKR/J cells have higher FC levels leading to CE/FC ratio ~ 3-fold higher in DBA/2J macrophages^4^. This phenotype appears to be due to effects on both FC esterification^5^, with the AKR strain having an N-terminally truncated *Soat1* gene, encoding the enzyme Acyl-coenzyme A: cholesterol acyltransferase 1 (ACAT1), and on CE hydrolysis^4^. We confirmed the role of autophagy-mediated lysosomal CE hydrolysis in BMDM, and demonstrated that autophagosome fusion with lysosomes is decreased in DBA/2J mice, resulting in impaired autophagic flux and inefficient CE clearance^4^.

In the present study, we applied quantitative trait locus (QTL) mapping to identify genes that impact lysosome function in BMDM derived from an AKR/J × DBA/2J F4 strain intercross. We discovered four macrophage lysosome function modifier (*Mlfm*) loci, with the strongest locus at the proximal region of chromosome 6 *(Mlfm1*). The *Mlfm1QTL* harbors the *Gpnmb* gene, encoding the glycoprotein nonmetastatic melanoma B (GPNMB). Our prior transcriptomic studies of AKR/J and DBA/2J BMDM found *Gpnmb* mRNA was significantly higher in AKR/J than DBA/2J, and that it’s expression was associated with its local genotype resulting in a *cis* expression QTL (eQTL)^6,7^. It was previously demonstrated that the DBA/2J strain carries a nonsense mutation in the *Gpnmb* gene^8^. Moreover, GPNMB has been shown to localize on autophagosome and lysosome membranes, and it promotes the recruitment of the autophagy protein light chain 3 (LC3) to the phagosome for lysosomal fusion^9^. *Gpnmb* expression is also upregulated in lysosomal storage diseases^10–12^, and in macrophages with inflammatory responses^13^. Here, we showed that knockdown of *Gpnmb* through siRNA in AKR/J macrophages, and *Gpnmb* deletion by CRISP/Cas9 in RAW 264.7 macrophages, decreased lysosomal function; while, an iso-congenic DBA/2 substrain (DBA/2J-Gpnmb + /SjJ) with the wild type *Gpnmb* gene increased lysosome function compared to DBA/2J BMDM. Therefore, our results validated *Gpnmb* as the causal lysosomal function modifier gene at the *Mlfm1* locus.

## Results

### DBA/2J macrophages has decreased lysosome function

**Figure 1:**
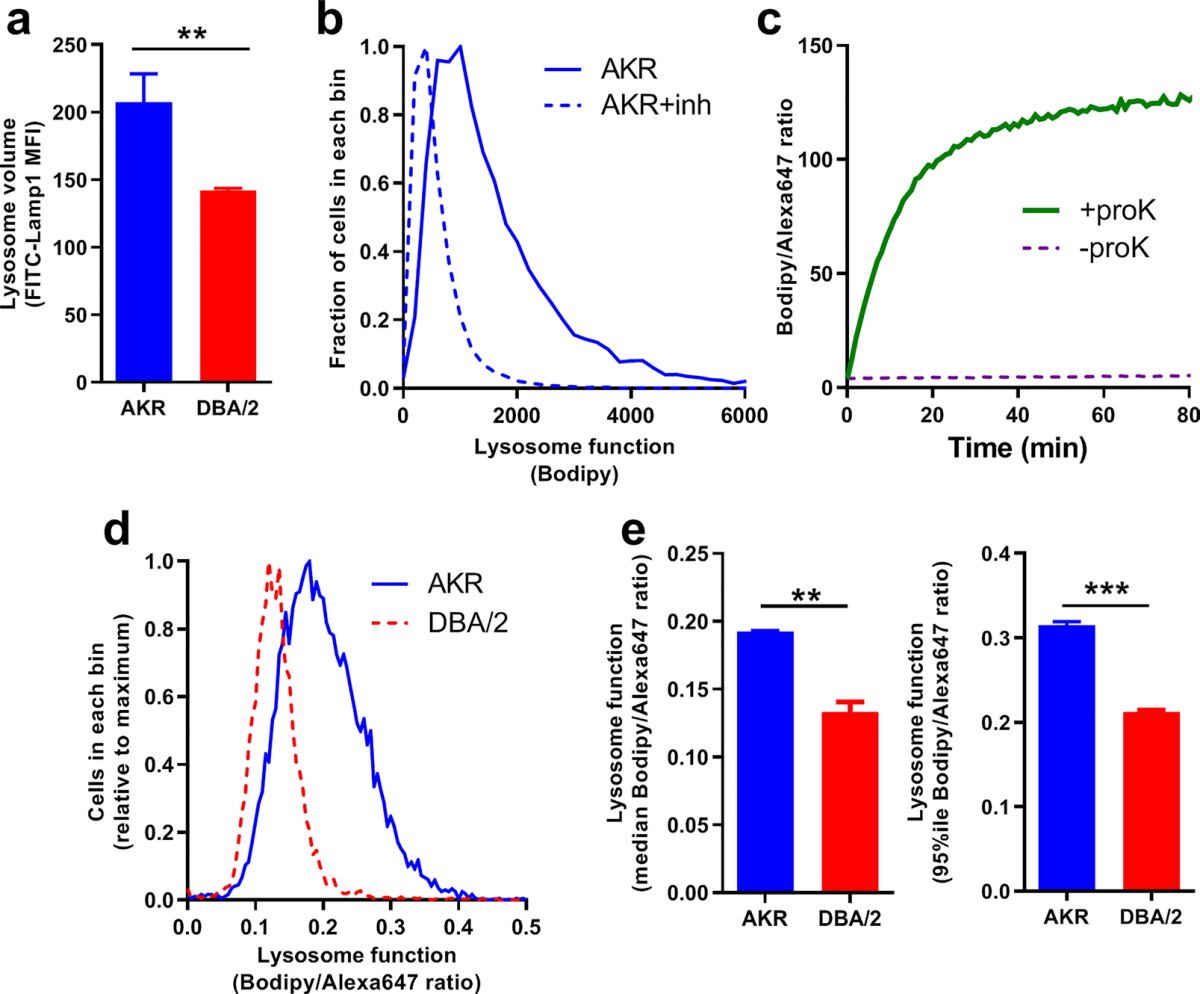
Decreased lysosome function in DBA/2J versus AKR/J macrophages. (**a**) Lysosome volume was assessed by incubating BMDM with 10 µg/mL FITC-labeled anti-Lamp1 antibody and examined by flow cytometry (median fluorescence intensity; n = 3 per strain, AKR blue bars; DBA/2 red bars). (**b**) Lysosome function in AKR/J BMDM was assessed by incubating cells with DQ-ovalbumin for 30 min and examined by flow cytometry (solid line). Lysosome inhibition with 3 h pretreatment of 10 µg/mL E64d plus 10 µg/mL pepstatin A led a leftward shift (dashed line) indicating decreased lysosome function. (**c**) Validation of lysosome function indicator in vitro showed proteinase K increased Bodipy/Alexa647 fluorescence ratio. (**d**) Lysosome function assay in AKR (solid blue line) and DBA/2 (dashed red line) BMDM was assessed by incubating cells with 2 µg/mL lysosome function indicator for 1 h and examined by flow cytometry. (**e**) Analysis of lysosome function in AKR (blue bars) and DBA/2 (red bars) BMDM using median (left panel) or the 95th percentile (right panel) fluorescence intensity ratio (duplicate assay) (**, *p* < 0.01; ***, *p* < 0.001 by two-tailed t-test). Graphs prepared using GraphPad Prism software v9.0.0 (www.graphpad.com).

DBA/2J versus AKR/J BMDM had less intense lysosomal staining as detected by immunofluoresence using a Lamp1 antibody (Supplemental Fig. 1). This was quantitatively confirmed by flow cytometry where DBA/2J versus AKR/J BMDM showed 32% decreased Lamp1 staining (*p* < 0.01, Fig. 1a). To investigate whether lysosome function is different in AKR/J and DBA/2J BMDM, we used a commercially available compound, DQ-ovalbumin, in which proteins are heavily labeled with Bodipy leading to fluorescence self-quenching. This compound is taken up by pinocytosis and accumulates in lysosomes, where proteolysis of DQ ovalbumin reduces the quenching, increasing Bodipy fluorescence^14^. We validated that cellular Bodipy fluorescence was blunted by pretreating cells with lysosomal protease inhibitors E64d and pepstatin A (Fig. 1b). However, this assay is also subject to the level of its cellular uptake, thus, we lightly labeled lysine residues on DQ ovalbumin with Alexa647, allowing us to measure the Bodipy/Alexa647 ratio as an indicator of lysosome function corrected for cellular uptake. This ratiometric lysosome function indicator was validated in vitro, showing that the Bodipy/Alexa647 ratio increased robustly by incubation with proteinase K (Fig. 1c). We next assessed lysosome function in AKR/J and DBA/2J BMDM by measuring the Bodipy/Alexa647 fluorescence ratio in each cell by flow cytometry and plotting the frequency distribution of this ratio in ≈10,000 cells (Fig. 1d). The median Bodipy/Alexa647 ratio was 45% higher in in AKR/J versus DBA/2J (*p* < 0.01, Fig. 1e left panel), while the 95th percentile was 49% higher in AKR/J versus DBA/2J (*p* < 0.001, Fig. 1e right panel); demonstrating decreased lysosome function in DBA/2J BMDM.

**Figure 2:**
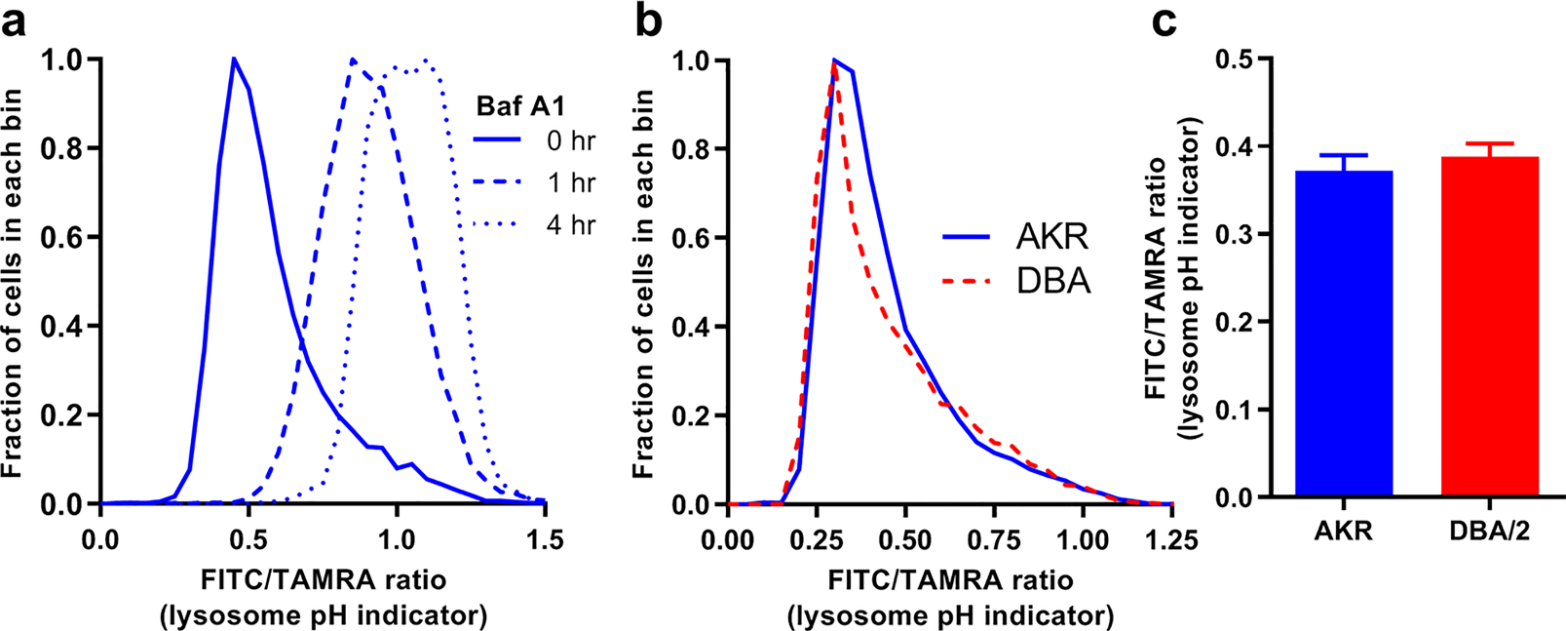
Unaltered lysosomal pH in DBA/2J versus AKR/J macrophages. (**a**) AKR BMDMs were incubated with 1 mg/mL FITC/TAMRA dextran for 18 h, and followed by a 4 h equilibration −/+ 10 µg Bafilomycin A1 added for the indicated times. Cells were analyzed by flow cytometry, demonstrating the effectiveness of this probe to assess lysosomal pH. (**b**) Lysosomal pH assay in AKR (solid blue line) and DBA/2 (dashed red line) BMDM was assessed by incubating cells with FITC/TAMRA dextran and analyzed by flow cytometry. (**c**) Analysis of lysosomal pH in AKR (blue bars) and DBA/2 (red bars) BMDM using median fluorescence intensity ratio (n = 3, not significant by two tailed t-test). Graphs prepared using GraphPad Prism software v9.0.0 (www.graphpad.com).

We then measured the lysosomal pH using commercially available FITC/TAMRA dual-labeled dextran, which is taken up by pinocytosis and accumulates in lysosomes. FITC fluorescence is pH-sensitive and is decreased as pH reduces in acidic organelles such as lysosomes, while TAMRA fluorescence is pH-insensitive. Thus, cellular FITC/TAMRA ratio is an indicator of lysosomal pH. We demonstrated the specificity of this assay by treatment with bafilomycin A1, a highly specific V-ATPase inhibitor, which led to increased FITC/TAMRA ratio in a time-dependent manner (Fig. 2a). However, there was no difference in FITC/TAMRA ratio comparing AKR/J and DBA/2J BMDM, indicating that lysosomal pH was not statistically different in those two strains (Fig. 2b,c). Therefore, we showed that DBA/2J versus AKR macrophages have decreased lysosome function, which cannot be attributed to increased lysosomal pH.

### Significant QTL for lysosome function maps to chromosome 6

**Figure 3:**
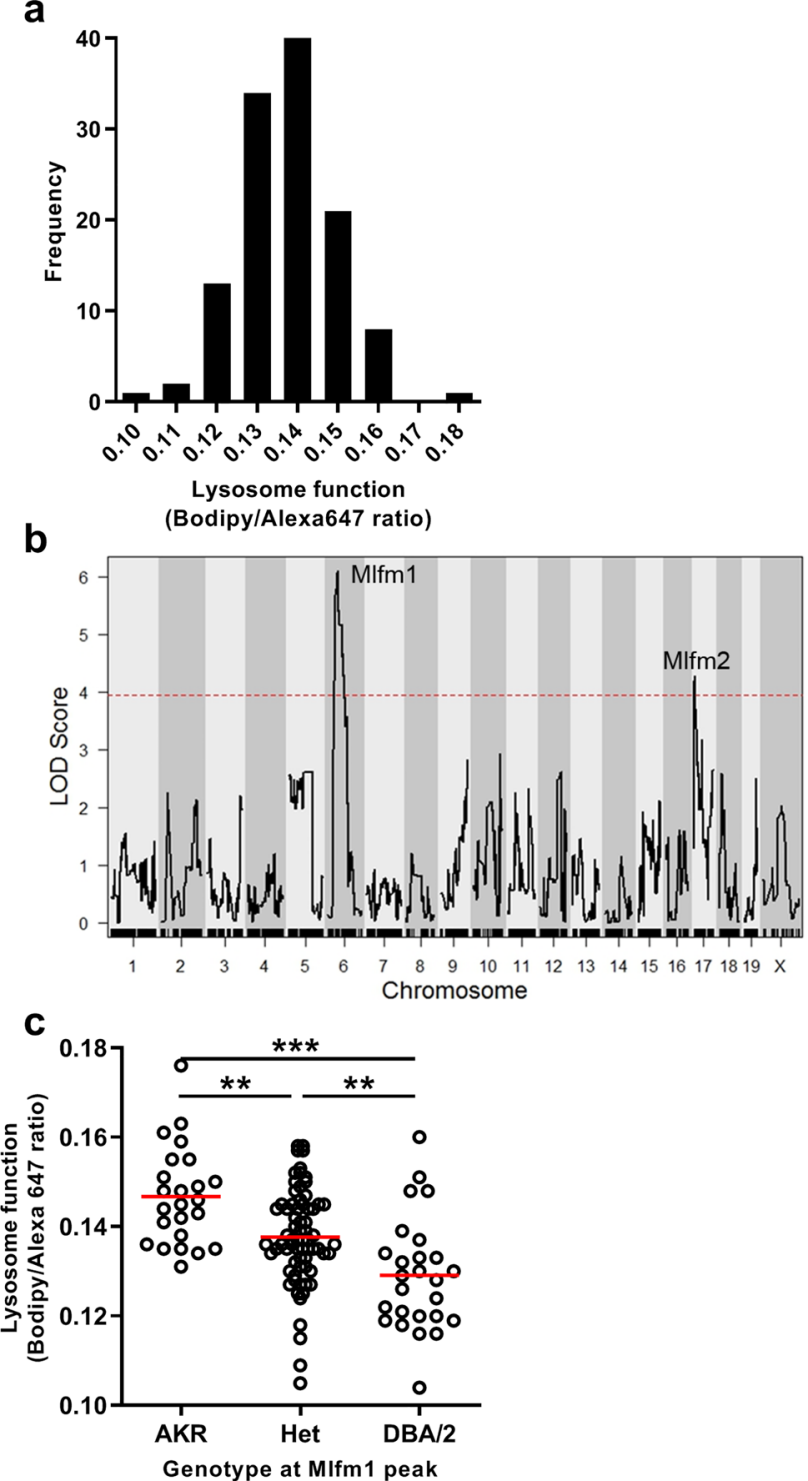
QTL analysis for lysosome function. (**a**) Normal distribution of the Bodipy/Alexa647 fluorescence ratio after F4 BMDM incubation with lysosome function indicator. (**b**) QTL LOD plot for lysosome function showing *Mlfm1* and *Mlfm2* on chromosomes 6 and 17, respectively. The dashed red line shows the genome-wide *p* = 0.05 threshold based on 10,000 permutations. (**c**) F4 BMDM lysosome function values by genotype at the *Mflm1* peak marker (mean values [red lines]; ANOVA linear trend test r^2^ = 0.208, *p* < 0.0001; **, *p* < 0.01, and ***, *p* < 0.001 by ANOVA Tukey posttest). QTL plot prepared using r/QTL^44^, other graphs prepared using GraphPad Prism software v9.0.0 (www.graphpad.com).

**Figure 4:**
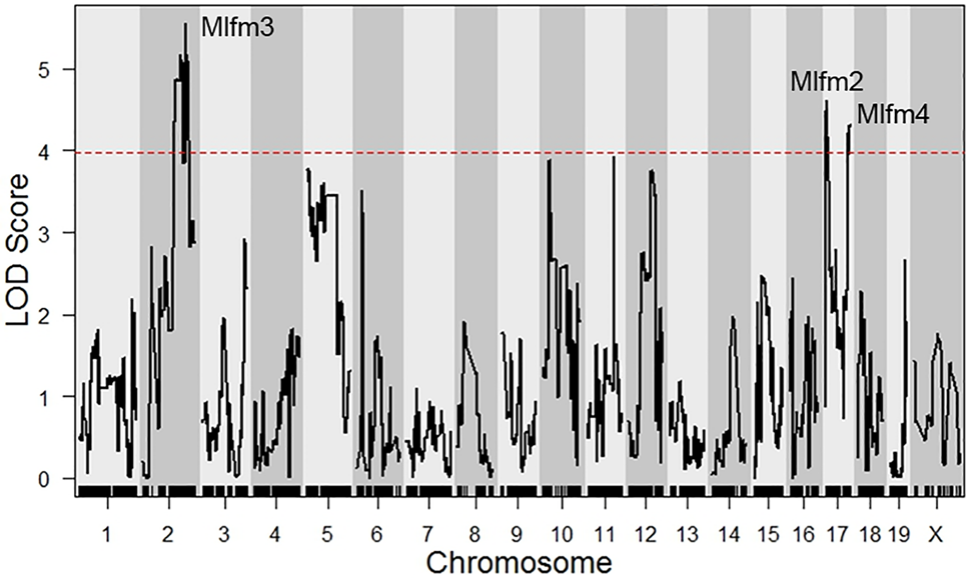
QTL mapping for lysosome function after adjusting for *Mlfm1* as an additive co-variate. The dashed red line shows the genome-wide *p* = 0.05 threshold based on 10,000 permutations. QTL plot prepared using r/QTL^44^.

We used a genetic approach to identify the gene region responsible for the strain difference in lysosome function. Lysosome function was measured in BMDM from 120 AKR/J × DBA/2J F4 mice by flow cytometry after incubation with the Bodipy/Alexa647 ovalbumin indicator; and, the Bodipy/Alexa647 ratio histograms were plotted for each BMDM from each F4 mouse. For genetic analysis we used the Bodipy/Alexa647 ratio 95th percentile values, as these were more significant than the median values (Fig. 1e). There was no significant effect of sex on this phenotype (*p* = 0.61), so we combined data from male and female BMDM. The lysosome function values for the F4 mice were normally distributed (Fig. 3a) and were used to perform QTL mapping. Two distinct macrophage lysosome function QTLs were identified on the proximal regions of chromosomes 6 and 17, and we named them macrophage lysosome function modifier (*Mlfm*) *Mlfm1* and *Mlfm2* (Fig. 3b). A similar LOD plot reproducing *Mlfm1* and *Mlfm2*, along with a new peak on the X chromosome, was obtained using the median Bodipy/Alexa647 ratio (Supplemental Fig. 2); however, the LOD peaks were not as significant, showing that the 95th percentile values, which may represent maximal lysosomal function from each F4 BMDM, yielded a better quantitative trait. The stronger *Mlfm1* QTL was located at 49.7 Mb on chromosome 6 (90% confidence interval 28.7–64.9 Mb), which had the logarithm of the odds (LOD) score of 6.09 (Table 1). There was a gene dosage effect of *Mlfm1* on lysosome function with each DBA/2J allele decreasing lysosome function by 6% (Fig. 3c, ANOVA linear trend test r^2^ = 0.208, *p* < 0.0001), indicating that this locus is associated with ~ 21% of the variance in lysosome function in the F4 cohort. *Mlfm2* was located at 9.5 Mb on chromosome 17 (90% confidence interval 6.0–16.5 Mb) with a LOD score of 4.28 (Table 1). After adjusting for the *Mlfm1* genotype as an additive covariate, new *Mlfm3* and *Mlfm4* loci were identified on the distal sides of chromosomes 2 and 17 (Fig. 4), with *Mlfm3* and *Mlfm4* LOD scores of 5.55 and 4.32, respectively (Table 2). *Mlfm2* on the proximal end of chromosome 17 was moderately strengthened after this adjustment (LOD score 4.61, Table 2).

**Table 1:** Macrophage lysosome function modifier QTLs.

QTL name	Chromosome	Peak Mb (90% confidence interval)	Max LOD	Genome wide *p* value
*lfm1*	6	49.7 (28.7–64.9)	6.09	< 0.05
*Mlfm2*	17	9.5 (6.0–16.5)	4.28	< 0.05

**Table 2:** Macrophage lysosome function modifier QTLs after adjusting for *Mlfm1*.

QTL name	Chromosome	Peak Mb (90% confidence interval)	Max LOD	Genome wide *p* value
*Mlfm2*	17	9.6 (6.0–17.1)	4.61	< 0.05
*Mlfm3*	2	147.5 (111.2–158.1)	5.55	< 0.05
*Mlfm4*	17	87.5 (79.4–89.1)	4.32	< 0.05

### Identification of *Gpnmb* as the gene responsible for the *Mlfm1* QTL

We next performed a Bayesian analysis for lysosome function at *Mlfm1* yielding a 90% confidence interval of 25.89 to 63.78 Mb, which contained 430 genes. Among them, *Gpnmb*, mapping at 48.99 Mb (0.17 Mb from the LOD peak), has a C>T mutation in DBA/2J mice leading to an early stop codon in exon 4^8^. This mutation is predicted to lead to non-sense mediated mRNA decay as traditionally defined^15^. We previously observed a DBA/2J-AKR/J strain difference in BMDM *Gpnmb* mRNA levels in a microarray study with DBA/2J macrophages expressing ~ 12.5 fold less *Gpnmb* mRNA^6^; and, our prior independent AKR/J × DBA/2J F2 strain intercross demonstrates a strong cis eQTL for *Gpnmb* expression in BMDM with a LOD score of 22^7^. *Gpnmb* codes for the GPNMB protein, which has been shown to be responsible for the recruitment of LC3 to the phagosome for lysosomal fusion^9^.

**Figure 5:**
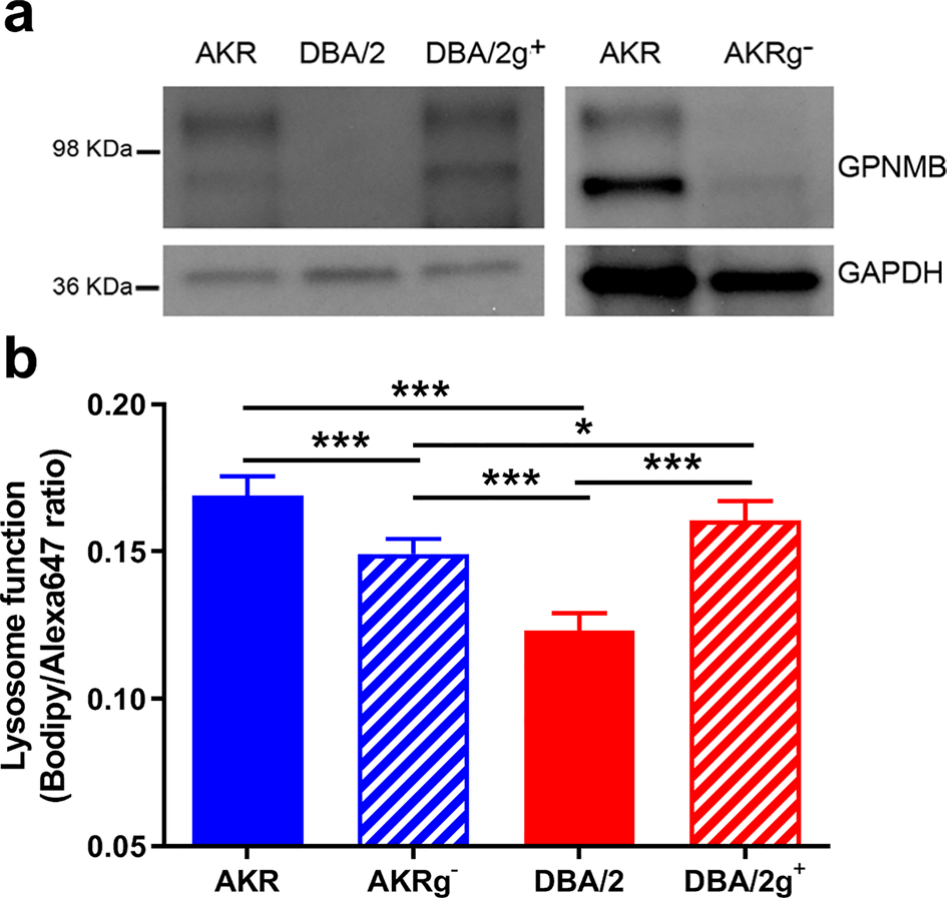
Altered lysosome function dependent upon *Gpnmb* expression. (**a**) GPNMB and GAPDH western blot from lysates of AKR, DBA/2, and DBA/2g + BMDM (left panel); and from AKR BMDM transfected with control or *Gpnmb* siRNA (right panel). Uncropped blots shown in Supplemental Figs. 3, 4. (**b**) Lysosome function was assessed by 1 h incubation of 2 µg/mL lysosome function indicator with BMDM from AKR control siRNA (solid blue bar), AKRg^-^ (striped blue bar), DBA/2 (solid red bar), and DBA/2g^+^ (striped red bar). Results were illustrated using median fluorescence intensity ratio (n = 5; *, *p* < 0.05; ***, *p* < 0.001 by ANOVA with Tukey posttest). Graph prepared using GraphPad Prism software v9.0.0 (www.graphpad.com).

**Figure 6:**
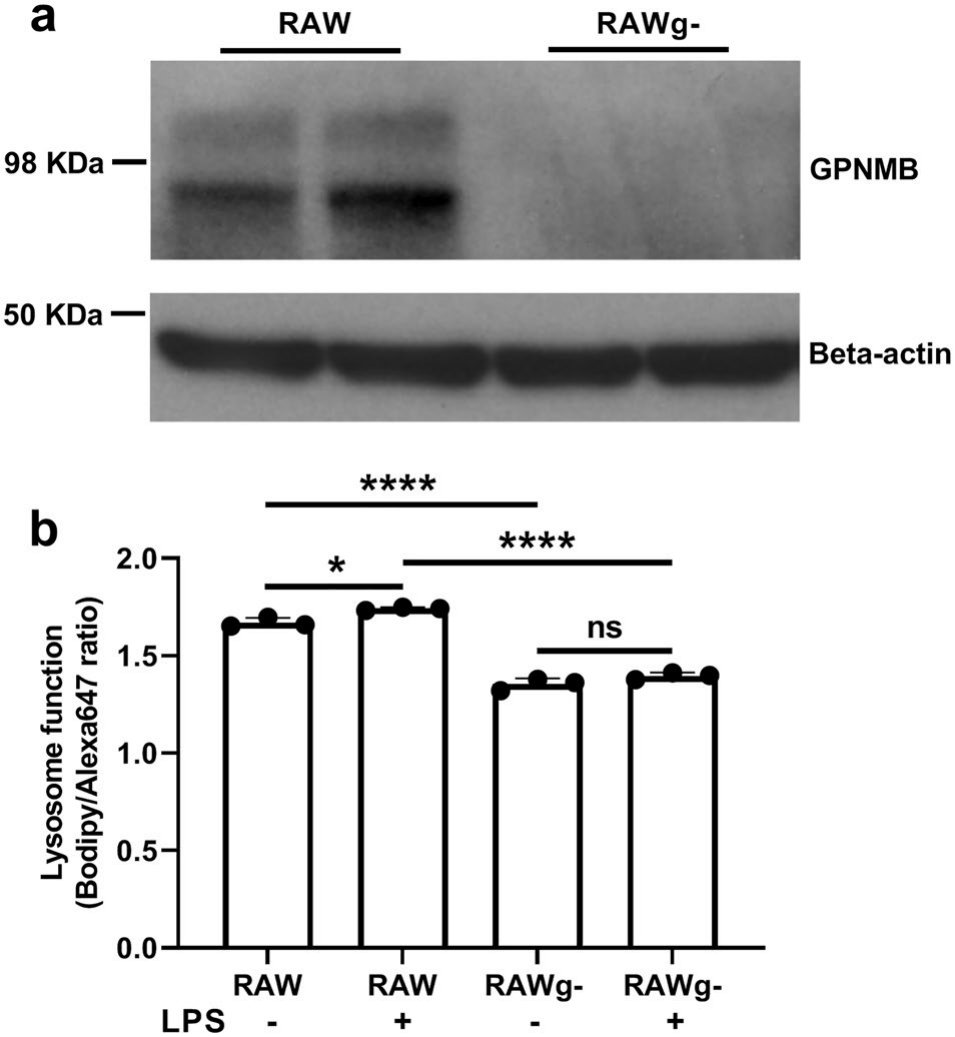
Decreased lysosome function in *Gpnmb* knockout RAW macrophages. (**a**) GPNMB western blot from lysates of RAW264.7 macrophages and *Gpnmb* knockout RAW264.7 macrophages. Uncropped blot shown in Supplemental Fig. 5. (**b**) Lysosome function was assessed by 1 h incubation of 2 µg/mL lysosome function indicator with RAW264.7 and *Gpnmb* knockout RAW264.7 macrophages with or without 24 h pretreatment with 250 ng/mL LPS. Results were illustrated using median fluorescence intensity ratio (*,*p* < 0.05; ****, *p* < 0.0001 by ANOVA with Tukey’s posttest). Graph prepared using GraphPad Prism software v9.0.0 (www.graphpad.com).

With *Gpnmb* being the strongest *Mlfm1* candidate modifier gene for lysosome function, we first performed Western blot in AKR/J and DBA/2J BMDM to confirm that there was no GPNMB expression detectable in DBA/2J macrophages (Fig. 5a). To determine whether the *Gpnmb* is responsible for the *Mlfm1* QTL for lysosome function, we used a DBA/2 coisogenic substrain, called DBA/2J-Gpnmb^+^/SjJ (referred to as DBA/2g^+^), which was separated from the main DBA/2J line before the *Gpnmb*^*R150X*^ null allele arose, and is backcrossed to the modern DBA/2J to maintain the wildtype *Gpnmb* allele on the DBA/2J background^16^. We confirmed GPNMB protein expression in this line by Western blot (Fig. 5a left side). Furthermore, we used siRNA to knockdown *Gpnmb* in AKR/J BMDM (referred to as AKRg^-^)_,_ with GPNMB protein expression decreased by 79% (Fig. 5a right side). We next measured lysosome function in AKR/J, AKRg^-^, DBA/2J and DBA/2g^+^ BMDM using the Bodipy/Alexa647 ovalbumin lysosome function indicator (Fig. 5b). We confirmed DBA/2J versus AKR/J BMDM had a 27% decrease in lysosome function (*p* < 0.001, by ANNOVA posttest). AKRg^-^ versus AKR/J BMDM had a 12% decrease in lysosome function (*p* < 0.001), and DBA/2g^+^ BMDM restored lysosome function to the comparable level of AKR/J BMDM (DBA2/g^+^ vs. DBA/2J, 30% increase, *p* < 0.001; DBA/2g^+^ vs. AKR/J, not significant). These findings indicate that the DBA/2J *Gpnmb* nonsense mutation allele is responsible for the *Mlfm1* QTL, and thus plays a major role in the AKR/J-DBA/2J strain effect on lysosome function. To replicate this effect in a different genetic background, we used CRISPR/Cas9 in the RAW264.7 macrophage cell line (referred to as RAW), derived from the BALB/c strain, to delete the entire *Gpnmb* gene (referred to as RAWg^-^). We confirmed lack of GPNMB protein expression in RAWg^-^ by Western blot (Fig. 6a). RAWg^-^ versus RAW macrophages had 19% decreased lysosome function (*p* < 0.0001, Fig. 6b). However, in the presence of LPS pretreatment, RAWg- versus RAW macrophages had 25% decreased lysosome function (*p* < 0.0001, Fig. 6b). These data confirm that *Gpnmb* expression increased lysosome function and supports our finding that *Gpnmb* was responsible for the *Mlfm1* QTL.

## Discussion

We previously crossed apoE-deficiency onto six inbred strains, DBA/2J, C57BL/6J, 129/SV-ter, AKR/J, BALB/cByJ, and C3H/HeJ; and, among these strains the DBA/2J has the largest aortic root atherosclerotic lesions, while AKR/J was one of several strains with small lesions^3^. This led us to follow up with two independent strain intercrosses to identify atherosclerosis modifier genes using the DBA/2J and AKR/J parental strains, which identified three confirmed atherosclerosis QTLs, *Ath22*, *Ath26*, and *Ath28*^7,17^. Since macrophages are a key cell type in atherogenesis, we also performed eQTL analysis of BMDM from these same two independent AKR/J × DBA/2J strains intercrosses in order to gain insights into potential atherosclerosis modifier candidate genes^7,18^. We also started series of studies to explore BMDM phenotypes from these two parental strains. Thus far we found significant strain effects on cholesterol ester loading, cholesterol efflux to apolipoprotein A1 or HDL acceptors, autolysosome formation^4^, and, in the present study, lysosome function. To identify QTL loci for these traits, we bred an F4 strain intercross and froze down aliquots of bone marrow for subsequent phenotype studies. We recently reported the first of these QTL studies, which identified an AKR/J deletion in exon 2 of the *Soat1* gene, encoding acyl-CoA:cholesterol acyl transferase 1, also known as ACAT1, as the strongest locus modifying cholesterol ester loading^5^.

The DBA/2J and AKR/J inbred strains have been useful in many areas of mouse physiology and disease. For example, the DBA/2J strain is susceptible to epicardiac calcification, which was mapped to the *Dyscalc1* QTL; and, the causal gene was identified *Abcc6* gene which has undetectable expression in the DBA/2J strain^19^. Another feature of the DBA/2J strain is that ~ 70% of these mice develop glaucoma by 12 months of age, after iris pigment dispersion (ipd) and iris stromal atrophy (isa)^16^. In a DBA/2J × C57BL/6J strain intercross, the ipd and isa phenotypes segregated to the *ipd* locus on chromosome 6 and the *isa* locus on chromosome 4^20^. Subsequent DBA/2J × CAST/Ei intercrosses fine mapped the *ipd* locus, and led to the discovery that this phenotype was due to a nonsense mutation in the *Gpnmb* gene (*Gpnmb*^*R150X*^) in the DBA/2J strain^8^. Furthermore, DBA/2 substrains with the wildtype *Gpnmb* gene do not have the ipd phenotype^8^. Thus, the same DBA/2J *Gpnmb* nonsense allele responsible for decreased macrophage lysosome function in our study is responsible for the ipd phenotype in the eye.

*Gpnmb* encodes for Glycoprotein Non-Metastatic Protein B (GPNMB) which was originally discovered in a melanoma cell line^21^. This protein, also called osteoactivin, DC-HIL, or hematopoietic growth factor inducible neurokinin-1, has been studied extensively in many contexts including cancer, kidney injury, obesity, non-alcoholic steatohepatitis, Parkinson disease, osteoarthritis, lysosome storage disorders, and heart failure; and, in most of these contexts expression of GPNMB is induced by the related pathology, likely in response to lysosomal stress^10,22–27^. However, loss of *Gpnmb* expression in DBA/2J mice is associated with preserved cardiac function after myocardial infarction^28^. The role of GPNMB/osteoactivin in bone formation, osteoblasts, and osteoclasts has been studied extensively. DBA/2 versus C57BL/6 mice have smaller femoral cross section area and study of an F2 cohort from intercrossing these two strains identified four gender-independent QTLs for this trait, including one on chromosome 6 where the *Gpnmb* gene resides^29^. Various transgenic models of GPNMB/osteoactivin over expression have been studied for effects on bone morphology and osteoclast number and function, with some discrepancy between non-tissue specific expression and osteoclast specific expression^30–32^. More relevant to the current study, DBA/2 and DBA/2g + mice have been assessed for bone phenotypes. DBA/2 versus DBA2g + mice have decreased femur bone cross sectional area/mass, marrow area, and cortical bone porosity associated with decreased osteoblast differentiation, proliferation, and survival, as well as with decreased osteoclast function, but increased osteoclast differentiation ex vivo^33,34^. Thus, there is much interest in *Gpnmb* and the role it plays in a multitude of diseases and in normal physiology.

In the current study, we verified that the *Gpnmb* null allele in the DBA/2J strain was likely responsible for the *Mlfm1* QTL on chromosome 6, the strongest locus for macrophage lysosome function. In addition to the *Mlfm1* QTL, we identified 3 other *Mlfm* loci on the distal region of chromosome 2 (*Mlfm3*) and on the proximal and distal regions of chromosome 17 (*Mlfm2* and *Mlfm4*). Further study will be needed to elucidate whether *Gpnmb*, or the genes at the other *Mlfm* QTLs, can account for the decreased autolysosome formation and lipid droplet turnover observed in DBA/2J macrophages.

We were able to identify the likely causal gene for the *Mlfm1* QTL without laborious breeding of congenic strains for fine mapping. However, it is possible that other genes affecting lysosome function may also map to *Mlfm1* QTL; but, the most parsimonious explanation is that the *Gpnmb* gene is responsible for this QTL. Our success in this endeavor was aided by several factors including the use of an F4 intercross cohort leading to more recombinations per chromosome and by performing a high density genome scan leading to a precise QTL peak. Another fortuitous factor was the availability of the DBA/2 iso-congenic substrain expressing wildtype *Gpnmb*. In our experience several phenotype assays performed in mice have high coefficients of variation. For example, fatty streak aortic root lesion areas in 16 week old chow diet-fed apoE-deficient mice on inbred background strains often yield coefficients of variation approaching 50%. This large phenotypic variation, due to either stochastic or subtle environmental differences, leads to decreased power to detect QTLs. In the current study, we used ex-vivo cell based assays, which had smaller coefficients of variation of ~ 10%, leading to better power for QTL mapping even with a smaller sample size compared to many mouse QTL studies. We call this ex vivo cell based approach ‘QTL in a dish’. One advantage of this method is the ability to treat cells with compounds or conditions that would be difficult to perform or painful in live mice. This ‘GWAS in a dish’ method is also being used to study phenotypes in different cell types derived from the differentiation of human induced pluripotent stem cells originating from cohorts consisting of ~ 100 to 200 subjects^35–37^.

## Methods

### Mouse strains

AKR/J, DBA/2J, and DBA/2J-Gpnmb^+^/SjJ (stock # 007048) mice were obtained from JAX. The DBA/2J-Gpnmb^+^/SjJ coisogenic mice were from the DBA/2 *Sandy* substrain, which was separated from the main DBA/2J line in the early 1980s, before the *Gpnmb*^*R150X*^ null allele was fixed in the DBA/2J stock. Modern backcrossing to DBA/2J mice was performed to maintain the wildtype *Gpnmb* (g^+^) allele on the DBA/2J background^16^. All mouse studies were approved by the Cleveland Clinic Animal Care and Use Committee, and all mouse studies were performed in accordance with the approved protocol. Mouse studies were carried out in compliance with the ARRIVE guidelines (http://www.nc3rs.org.uk/page.asp?id=1357).

### Generation and genotyping of AKR/J × DBA/2J F4 mice

Parental male AKR/J and female DBA/2J mice were crossed to generate the F1 generation, fixing the Y chromosome from the AKR/J strain. Two breeding pairs of F1 mice were bred to generate the F2 mice, and two breeding pairs of F2 mice were used to generate F3 mice. Six breeding pairs of F3 mice were used to generate the 122 F4 mice, which consisted of 70 males and 52 females^5^. Healthy F4 mice were sacrificed at 8–10 weeks of age. Ear tissue was collected from each mouse and digested overnight at 55 °C in lysis buffer containing 20 mg/mL proteinase K. DNA was ethanol precipitated and resuspended in 10 mM Tris 1 mM EDTA (pH = 8). Femurs were promptly flushed after sacrifice, and bone marrow cells were washed, aliquoted, and cryopreserved. Cells were thawed and differentiated into macrophages at the time of experimentation, as described below. F4 mice were genotyped as described previously^5^. Briefly, the GeneSeek MegaMUGA SNP array was used, and filtering for call frequency and strain polymorphism using parental and F_1_ DNA yielded 16,975 informative SNPs that were used for QTL mapping. All marker locations are based on NCBI Mouse Genome Build 37.

### Bone marrow-derived macrophages

Bone marrow derived macrophages were obtained from female and male F4 mice (two were excluded due to low yields) and female mice on the AKR/J, DBA/2J and DBA/2g^+^ background. Bone marrow cells were suspended in macrophage growth medium (DMEM, 10% FBS, 20% L-cells conditioned media as a source of MSCF) as previously described^38,39^ and plated in tissue culture coated 6, 12, or 24 well plates. The media was renewed twice per week. Cells were used for experiments 10 to 14 days after plating when the bone marrow cells were confluent and fully differentiated into macrophages. When required, AKR/J cells were transfected with 50 nM silencer-select *Gpnmb* (4390771, Thermofisher Scientific) or control (4390843, Thermofisher Scientific) siRNA using TransIT-TKO (MIR2150, Mirus) as described by the manufacturer. Cells were incubated with the siRNA complexes for 48 h, media was then replaced with fresh macrophage growth media (in the presence or absence of 50 µg/mL AcLDL as indicated), and incubated for another 24 h before experiments.

### Lipoprotein preparations

Human LDL (1.019 < d < 1.063g/mL) were prepared by ultracentrifugation from de-identified expired blood bank human plasma (reviewed by the Cleveland Clinic Institutional Review Board and found exempt from human research rules). LDL was acetylated as described previously^40,41^ and dialyzed against PBS with 100 µM EDTA and 20 µM BHT. Protein concentrations of lipoproteins were determined using an alkaline Lowry assay^42^. When indicated, cells were loaded with 50 µg/mL of AcLDL for 24 h.

### Lysosome volume assay

Macrophages were first stained and gated for live cells with LIVE/DEAD Fixable Blue Dead Cell Stain (L23105, Thermofisher Scientific). The cells were fixed in 4% paraformaldehyde and permeabilized with saponin, lysosomes were labeled using 10 µg/mL dilution of FITC-labeled antibody against mouse Lamp-1 (ab24871, abcam), a lysosomal structural protein, and detected by flow cytometry.

### Lysosome function assay

To validate the use of DQ-ovalbumin as a surrogate measure of lysosome function, cells were pre-treated for 3 h in absence or presence of 10 µg/mL E64d (E8640, Sigma-Aldrich) and 10 µg/mL pepstatin A (P5318, Sigma-Aldrich) before incubating for 30 min with the reagent. To make lysosome function indicator, 1 mg of DQ-ovalbumin (D12053, Thermofisher Scientific) in 0.1 M sodium bicarbonate was incubated with 98 µg of Alexa Fluor 647 succinimidyl ester (A20006, Thermofisher Scientific) for 1 h at room temperature (3:1 dye:protein mole ratio). The reaction was stopped by incubating the conjugate with 0.1 mL of 1.5 M hydroxylamine (pH 8.5) for 1 h at room temperature. The conjugate was purified by extensive dialysis. To evaluate lysosome function, macrophages were incubated with 2 µg/mL of lysosome function indicator for 1 h, washed with PBS, and suspended using CellStripper (25056CI, Corning). To evaluate lysosomal pH, cells were incubated for 18 h with 1 mg/mL FITC-TAMRA dextran (D1951, Thermofisher Scientific) followed by a 4 h chase period in absence or presence of 10 µM Bafilomycin A1 (B1793, Sigma-Aldrich) for the indicated times. In all experiments, 10,000 cells were analyzed by flow cytometry with a LSRII device (BD) using the following lasers and filters: 488 nm excitation and 515/20 nm emission (FITC and Bodipy), 639 nm excitation and 660/20 nm emission (Alexa647) and 532 nm excitation and 575/26 nm emission (TAMRA). Flowjo v10.6.1 software (www.flowjo.com) was used to export data for each cell for ratiometric analyses.

### CRISPR/Cas9 *Gpnmb* knockout

RAW264.7 macrophages (obtained from ATCC, # TIB-71) were cultured in DMEM supplemented with 10% FBS. In order to generate a *Gpnmb* knockout cell line we designed two sgRNAs using CRISPOR^43^ that spanned the entire *Gpnmb* gene, such that excision between their target sites would lead to a 21.9 kb deletion (Table 3). 1 × 10^6^ RAW264.7 cells were co-transfected via nucleofection (Amaxa) with the two mouse *Gpnmb* sgRNAs (Synthego, 0.6 nM each), which were pre-incubated with 0.07 nM Cas9 protein (Synthego). Transfected cells were plated in 96-well dishes to approximately 1 cell/well then clonally expanded. Clones were screened via Western Blot to find cells without GPNMB protein expression.

**Table 3:** sgRNAs used to delete the mouse *Gpnmb* gene.

sgRNA	Sequence	Position on Chr. 6
*mu*sg*Gpnmb* #1	ACCAACGACCAGGUUUCGUU	49,036,355–49,036,374
*mu*sg*Gpnmb*#2	GUGCATCGCCUUCAAACUAU	49,058,250–49,058,269

### Western blot

AKR/J, AKRg^-^, DBA/2J, DBA/2g^+^ macrophages, and RAW macrophages with or without CRISPR/Cas9 *Gpnmb* editing were lysed in RIPA buffer and equal protein levels loaded on 4–20% tris-glycine gels. After transfer, membranes were probed with antibodies against GPNMB (AF2330, R&D Systems) and GAPDH (FL-335, Santa Cruz).

### QTL mapping analysis

QTL mapping of macrophage lysosome function (*Mlfm*) from 120 out of 122 AKR/J × DBA/2J F4 BMDMs (the other 2 lines did not yield a sufficient number or viable cells) was performed using R/qtl software, with the final genotype and phenotype data formatted for analysis in the Supplemental Table S1 (95^th^ percentile values) and Table S2 (median values)^44^. The “scanone” function was utilized using Haley-Knott regression by specifying the “method” argument as “hk”. Genome-wide p-values were ascertained via permutation analysis, using 10,000 permutations by specifying the “n.perm” argument in the “scanone” function. QTL 90% confidence intervals were calculated using the 1-LOD drop off method. The credible interval for the *Mlfm1* locus was also determined by using the Bayesian credible interval (“bayesint”) function in R/qtl, with the “prob” argument set at 0.95. Since *Mlfm1* had a significantly higher peak LOD score than any other locus, QTL mapping was performed using the genotype from the best associated *Mlfm1* marker as an additive covariate (“addcovar”) in the “scanone” function of R/qtl. The *Mlfm1* corrected data were subjected to 10,000 permutation analyses to determine genome-wide p-values. To aid in prioritizing candidate genes, a custom R function termed “flank_LOD” was written (http://www.github.com/BrianRitchey/qtl). This “flank_LOD” function utilizes the “find.flanking” function in R/qtl and returns the LOD score of the nearest flanking marker for a given candidate gene position based on “scanone” output data. Genes in a QTL interval were determined by custom written R functions (“QTL_gene” and “QTL_summary”) which utilized publicly available BioMart data from Mouse Genome Build 37. A custom written R function (“pubmed_count”) which utilized the rentrez package in R was used to determine the number of PubMed hits for Boolean searches of gene name and terms of interest. Custom written R functions (“sanger_AKRvDBA_missense_genes” and “missense_for_provean”) were used to determine the number of non-synonymous mutations between AKR/J and DBA/2J in QTLs, as documented by the Wellcome Trust Sanger Institute’s Query SNP webpage for NCBIm37 (https://www.sanger.ac.uk/sanger/Mouse_SnpViewer/rel-1211). Custom written VBA subroutines (“Provean_IDs” and “Navigate_to_PROVEAN”) were used to automate PROVEAN software (http://provean.jcvi.org/seq_submit.php) queries for functional effects of missense mutations in each QTL, with rentrez functions utilized to retrieve dbSNP and protein sequence data. Ultimately, custom R code was used to generate output tables. Deleterious mutations were designated as defined by PROVEAN parameters^45^. Custom code can be found at http://www.github.com/BrianRitchey/qtl.

### Statistics

Large data sets were tested for normal distributions and passed, thus parametric statistics were used. Comparison of two conditions was performed by two-tailed student t-test, and comparison of multiple conditions was performed by ANOVA with Tukey or linear trend posttest. All data are shown as mean ± S.D. Statistics were performed using GraphPad Prism software v9.0.0 (www.graphpad.com).

### Ethics approval

All mouse studies were approved by the Cleveland Clinic Institutional Animal Care and Use Committee.

## Supplementary Information

Supplementary Information.

Supplementary Table S1.

Supplementary Table S2.

## Data Availability

All primary data from this work is available from the corresponding author upon request**.**

## Code availability

Custom R code can be found at http://www.github.com/BrianRitchey/qtl.
