# Supplementary material for: Quantitative trait locus mapping identifies the Gpnmb gene as a modifier of mouse macrophage lysosome function
Source: Sci Rep. 2021 May 13;11:10249. doi: 10.1038/s41598-021-89800-5 (PMC8119501; doi:10.1038/s41598-021-89800-5)
Supplement: Supplementary file 1 — Supplementary Information. [file 41598_2021_89800_MOESM1_ESM.pdf]

Quantitative Trait Locus Mapping Identifies the *Gpnmb* Gene as a Modifier of Mouse Macrophage Lysosome Function

Peggy Robinet[1,#$], Brian Ritchey[1$], Shuhui Wang Lorkowski[1$], Alexander M. Alzayed[1], Sophia DeGeorgia[1], Eve Schodowski[1], C. Alicia Traughber[1,2], Jonathan D. Smith[1,2,*]

[1] Department of Cardiovascular & Metabolic Sciences, Lerner Research Institute, Cleveland Clinic, Cleveland, OH, USA, 44195

[2] Department of Molecular Medicine, Cleveland Clinic Lerner College of Medicine of Case Western Reserve University, Cleveland, OH, USA 44195

* Corresponding author, email: smithj4@ccf.org, ORCID ID 0000-0002-0415-386X.

[#] Current address: ProEd Communications, Inc., Beachwood, OH, USA 44122

[$] These authors contributed equally to this work

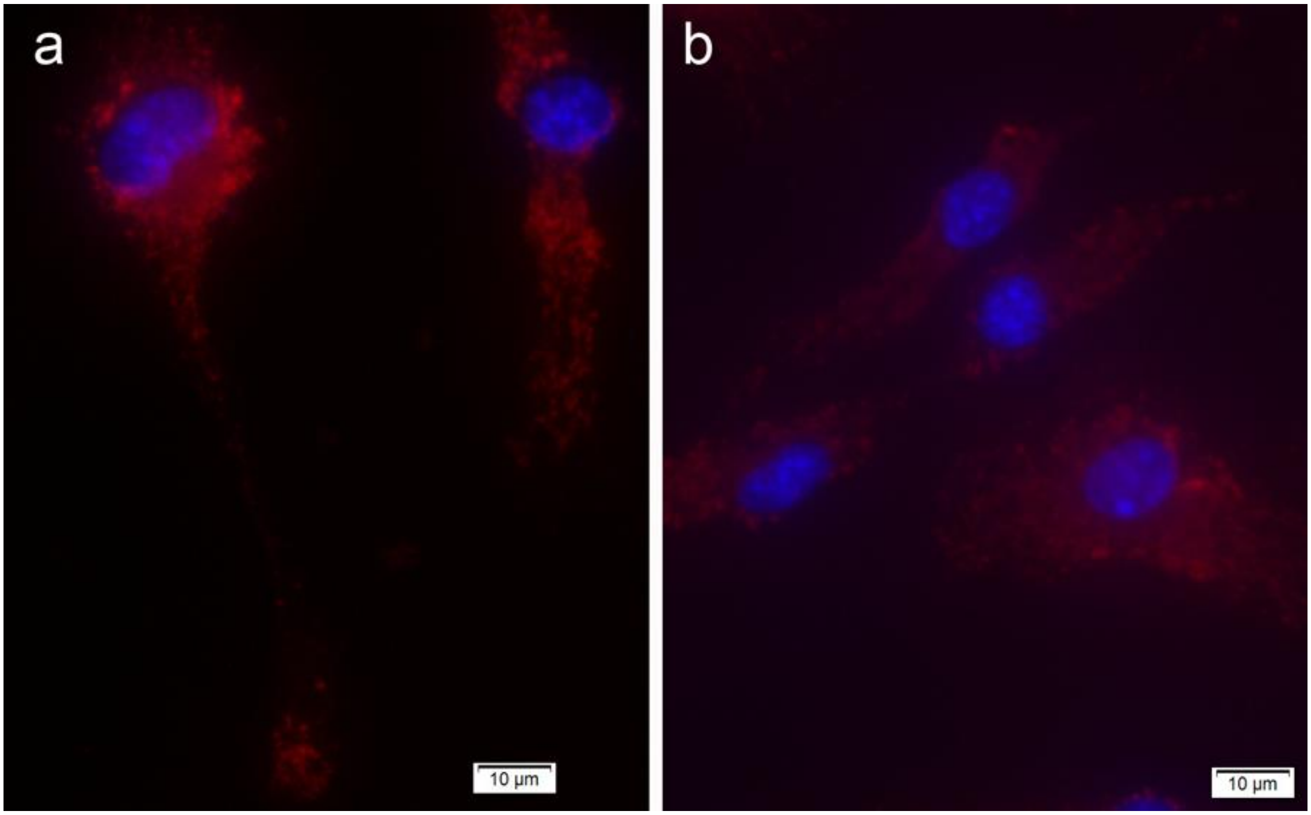

Supplemental Fig. 1. Lamp 1 immunostaining of AKR/J (a) and DBA/2J (b) BMDM shown in red, with DAPI-stained nuclei shown in blue. 60x objective lens.

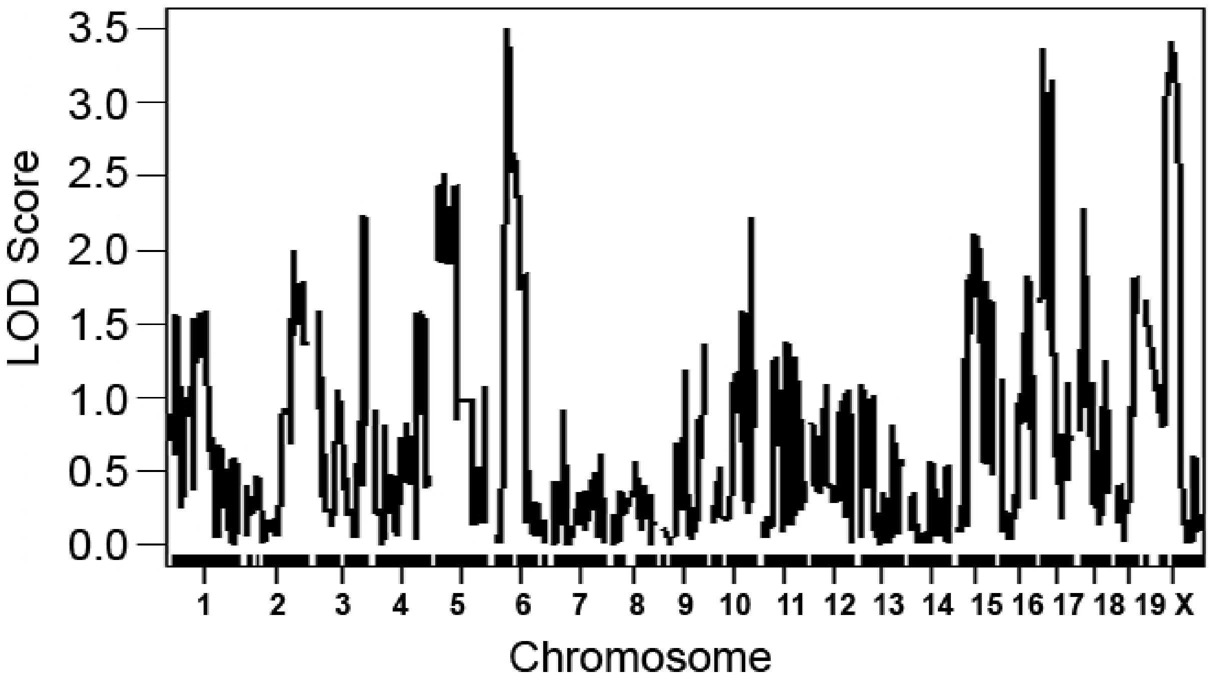

QTL name	Chromosome	Peak Mb (90% confidence interval)	Max LOD
Mlfm1	6	37.6 (28.7 – 69.6)	3.50
Mlfm2	17	8.3 (6.0 – 39.2)	3.36

Supplemental Fig. 2. LOD plot of lysosome function based on the median Bodipy/Alexa647 ratios for BMDM from 120 F4 mice. Data used in rQTL to perform this analysis is provided in Supplemental Table S2.

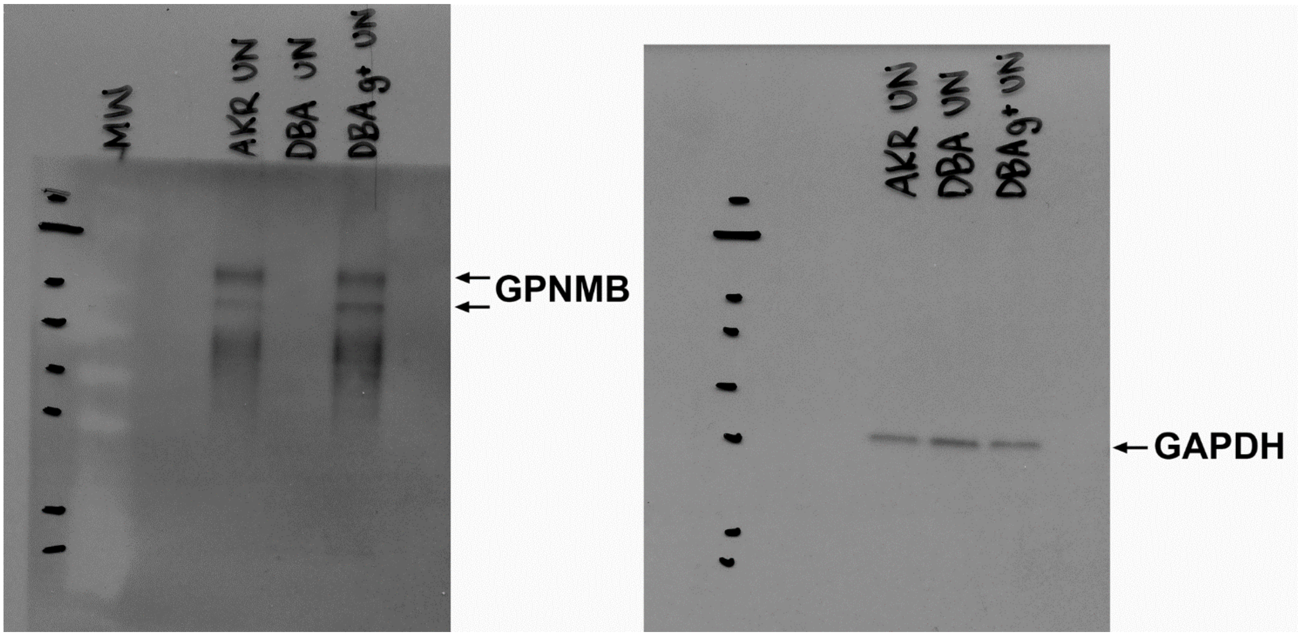

Supplemental Fig.3. Uncropped western blot probed for GPNMB(left panel) and GAPDH (right panel) used in Fig 5a, left side.

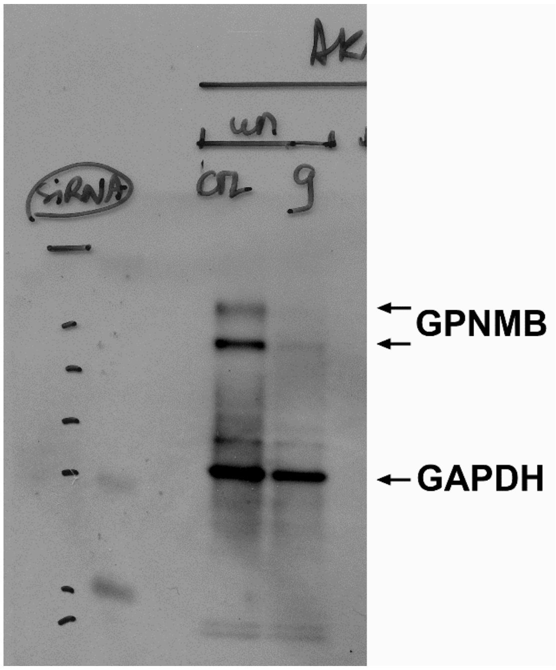

Supplemental Fig. 4. Uncropped western blot probed for GPNMB and GAPDH used in Fig 5a, right side.

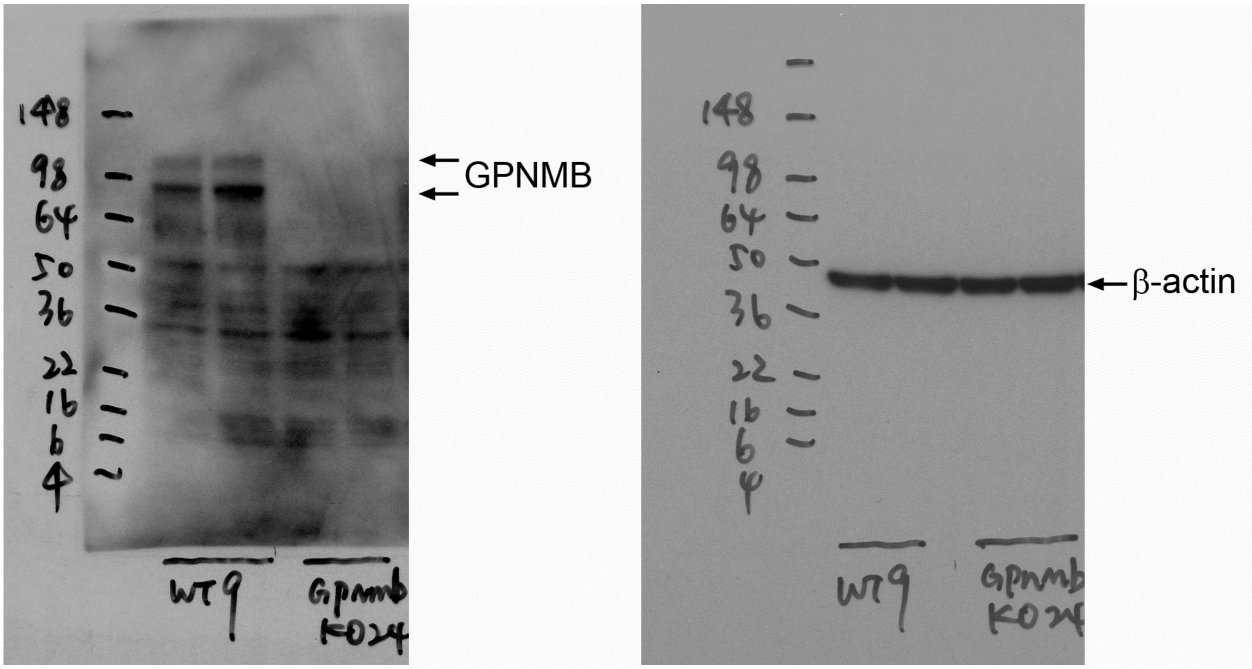

Supplemental Fig. 5. Uncropped western blot probed for GPNMB (left panel) and β-actin (right panel) used in Fig 6b.

Supplemental Table Captions (for separate csv files)

Table S1. Lysosome function phenotypes (95th percentile values) and genotypes formatted for r/QTL analysis, used for generating Figure 3/

Table S2. Lysosome function phenotypes (median values) and genotypes formatted for r/QTL analysis, used for generating Supplemental Figure 1.
